# Hepatic failure in a patient with rheumatoid arthritis treated with methotrexate: A case report

**DOI:** 10.1097/MD.0000000000032711

**Published:** 2023-01-27

**Authors:** Masayuki Miyata, Sho Ishiwata, Masahito Kuroda, Kazuhiro Tasaki, Kiyoshi Migita, Hiromasa Ohira

**Affiliations:** a Center for Rheumatology and Collagen Diseases, Fukushima Red Cross Hospital, Fukushima, Japan; b Department of Pediatrics, Fukushima Medical University School of Medicine, Fukushima, Japan; c Department of Gastroenterology, Fukushima Red Cross Hospital, Fukushima, Japan; d Department of Pathology, Fukushima Red Cross Hospital, Fukushima, Japan; e Department of Rheumatology and Collagen Diseases, Fukushima Medical University School of Medicine, Fukushima, Japan; f Department of Gastroenterology, Fukushima Medical University School of Medicine, Fukushima, Japan.

**Keywords:** fibrosis-4 index (FIB-4), hepatic failure, metabolic syndrome, methotrexate (MTX), nonalcoholic steatohepatitis (NASH), rheumatoid arthritis (RA)

## Abstract

Chronic liver damage from methotrexate (MTX) is not uncommon, and fatal outcome is rare. We experienced a case of hepatic failure leading to death. We considered the cause of death through this case and proposed a method to prevent the progression of this liver injury.

**Patient concerns:**

We report the case of a patient with rheumatoid arthritis treated with MTX for 15 years.

**Diagnosis, interventions, and outcomes:**

A liver biopsy revealed histological changes similar to those of advanced nonalcoholic steatohepatitis (NASH), most likely induced by MTX. MTX was discontinued after 4 years. Two years after the discontinuation, the patient died of irreversible hepatic failure. Her obesity, complicated by type 2 diabetes mellitus, might have aggravated MTX-induced NASH-like liver injury.

**Conclusion:**

Early diagnosis and immediate MTX discontinuation following NASH diagnosis and strict type 2 diabetes mellitus control might have prevented the irreversible progression of liver injury.

## 1. Introduction

Recently, various biological disease-modifying antirheumatic drugs and Janus kinase inhibitors have been developed and used to treat rheumatoid arthritis (RA). However, methotrexate (MTX) has long been used worldwide as an anchor drug for the treatment of RA because it is effective and affordable. Myelosuppression, interstitial pneumonia, and infection have attracted attention as major causes of death owing to MTX treatment. The most common side effect of MTX is hepatotoxicity.

Recently, a patient with RA who was treated with MTX died of hepatic failure at our hospital. She had obesity with type 2 diabetes mellitus (T2DM). The results of the histological examination of this patient was similar to those of nonalcoholic steatohepatitis (NASH). We have explained why she developed liver failure in the Discussion section. Her chronic liver injury was not diagnosed through routine liver function tests in the initial stages, which could have resulted in the progression of liver damage to an irreversible stage. We noticed a decrease in her blood platelet count (PLT); however, the cause of thrombocythemia was unclear. Subsequently, we retrospectively calculated the fibrosis-4 index (FIB-4) score as (age × aspartate aminotransferase [AST]/ PLT × √alanine aminotransferase [ALT])^[1,2]^ to evaluate the degree of chronicity of liver injury. The score increased significantly 4 years after the initiation of MTX treatment, indicating that she might have developed a chronic liver injury. This occurred 5 years before the liver biopsy and 11 years before her death (year of the patient’s death [X]−11). After this patient, we have treated >30 other patients with MTX-induced chronic liver injury. None of them died because of MTX hepatotoxicity. Therefore, we report the diagnosis of MTX-induced chronic liver injury and treatment of hepatotoxicity to prevent adverse outcomes. The procedures were conducted following the Declaration of Helsinki, and all investigations were approved by the institutional review board of the Fukushima Red Cross Hospital. Furthermore, written informed consent was obtained from the patient’s family members.

## 2. Case report

**Figure 1.**
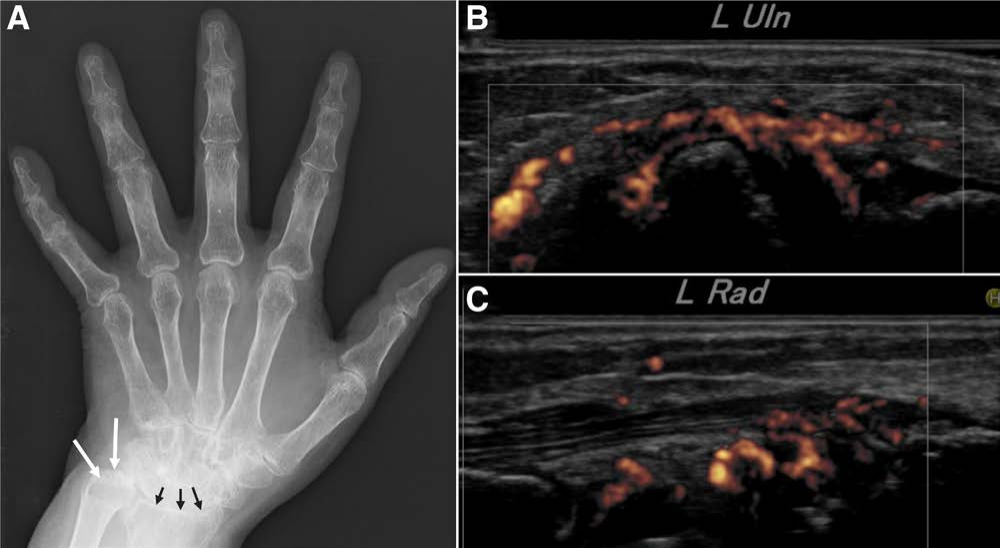
X-ray and US findings of the left hand. (A) X-ray of the left hand shows narrowing of the wrist joint (black arrows) and destruction of the ulnar styloid process (white arrows). Peripheral ulnar deviation from the metacarpophalangeal joint at 5th finger. Narrowing of the distal interphalangeal joints due to osteoarthritis was observed. US showed inflammation of the wrist joint on the (B) ulnar and (C) radial side. US = ultrasonography.

A 54-year-old woman with polyarthritis visited our outpatient department 15 years before her death (X−15). She had a clinical history of psoriasis; however, she had no rashes during her first visit. The patient had obesity (body mass index, 29.7 kg/m^2^; height, 148 cm; weight, 65 kg) that was complicated by T2DM and hypertension. She smoked 15 cigarettes daily but did not consume alcohol. The clinical features of her arthritis, such as the symmetrical distribution of lesions and X-ray and ultrasound findings, were similar to those of RA (Fig. 1). Blood tests, including tests for inflammatory response, liver function, renal function, rheumatoid factor, and anti-cyclic citrullinated peptide antibody, were performed. Rheumatoid factor and anti-cyclic citrullinated peptide antibody tests were negative. Thus, the patient was diagnosed with seronegative RA.

Treatment was initiated with 8 mg of MTX weekly. However, arthritis did not subside. Hence, intravenous administration of 3 mg/kg infliximab once daily for 2 to 8 weeks was added. The polyarthritis was initially well-controlled; however, there was a relapse after infliximab treatment, which was later replaced with subcutaneous administration of 50 mg etanercept weekly.

**Figure 2.**
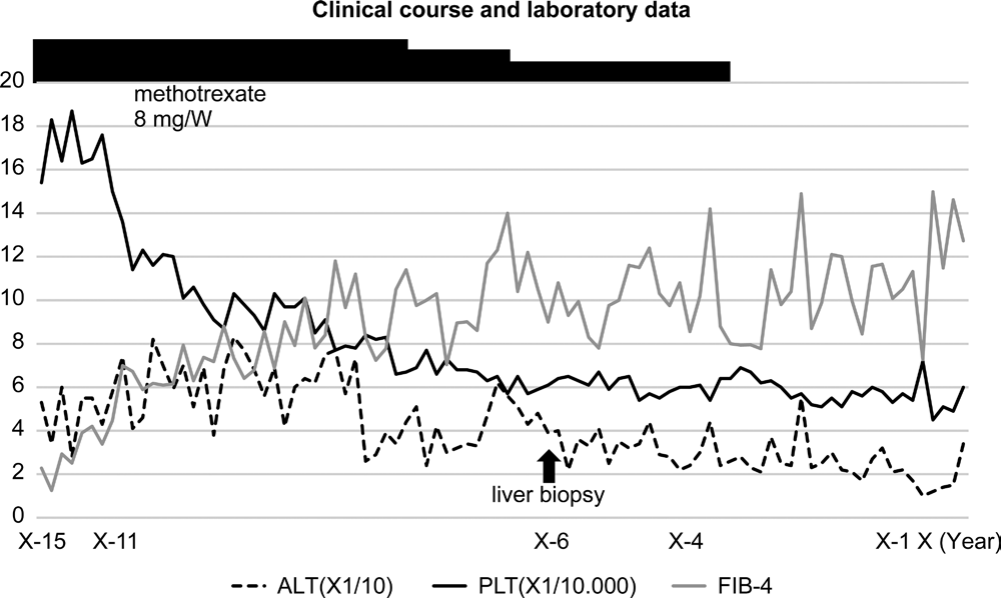
Laboratory data. The aspartate aminotransferase (AST)/alanine aminotransferase (ALT) ratio and platelet count (PLT) were 44/53 U/L and 15.4 × 10^4^/μL, respectively, at the time of initiating methotrexate (MTX) treatment on September 17, X−15. The fibrosis-4 index (FIB-4) at that time was 2.28. The FIB-4 index increased to 6.15, 4 years later, on November 16, X−11. PLT decreased gradually to 6.1 × 10^4^/μL, and the FIB-4 index increased to 9.0 at the time of liver biopsy. MTX discontinuation did not affect the FIB-4 index score. ALT = alanine aminotransaminase, FIB-4 = fibrosis-4 index, PLT = platelet count, X = year of the patient’s death.

We noticed a gradual decrease in her PLT from the initial 15.4 × 10^4^/μL to 6.1 × 10^4^/μL at 9 years after initiating MTX (X−6) (Fig. 2). A review of her clinical course showed that the PLT had decreased 4 years after initiating MTX (X−11) and it gradually decreased further. Her liver function was almost normal (AST/ALT = 52/39 U/L); however, she developed facial spider hemangioma and palmar erythema. Furthermore, an enlarged liver was observed in the epigastric region on palpation. Based on the negative results of platelet-associated immunoglobin G and these clinical findings, the patient developed a chronic liver injury resulting in thrombocytopenia.

**Figure 3.**
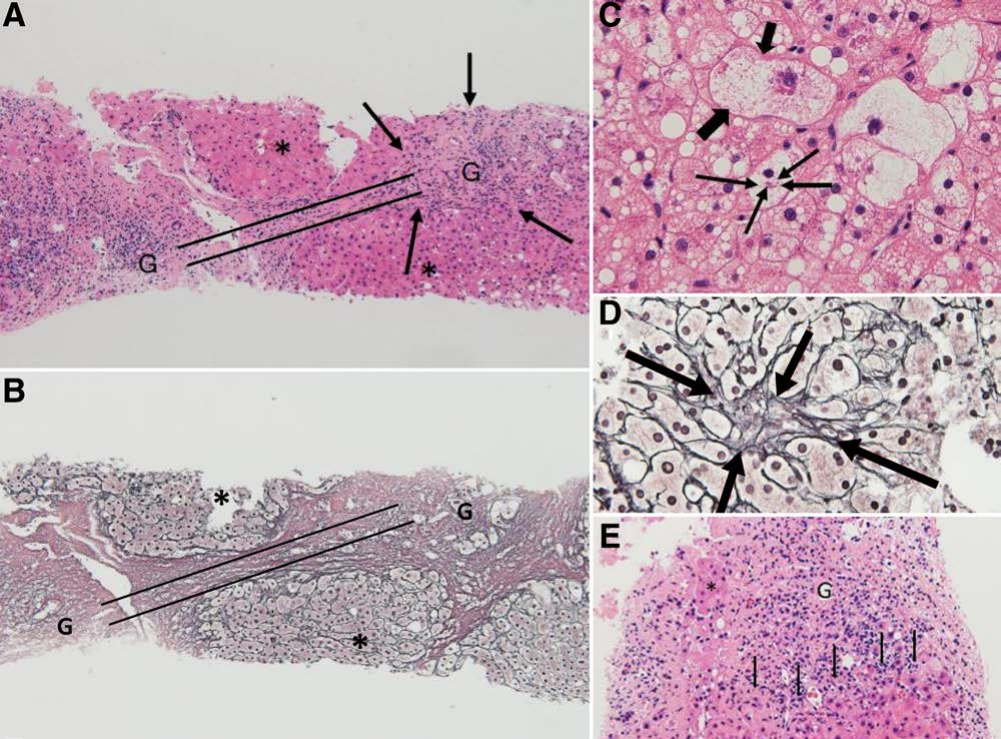
Histological examination of the liver on April 11, X−6. Hematoxylin-eosin staining (A: ×100, B: ×400) showed (A) extensive fibrosis and excessive inflammatory cell infiltration (thin arrows) in the portal vein area (G), bridging lobular distortion (parallel line shows bridging formation) or pseudolobular formation (*). (B) Marked ballooning (thick arrows) and Mallory–Denk bodies (thin arrows) of hepatocytes. Silver staining (C: ×100, D: ×400) showed (C) clear fibrosis with strong expansion at bridging formation between G and G, as shown by the parallel line; (D) hepatic pericellular fibrosis (thin arrows) even with weak expansion. (E) Hematoxylin-eosin staining (×400) showed lymphocyte infiltration (thin arrows) at the hepatic lobular margin bordering the portal zone (G) (interface hepatitis). This caused fibrotic enlargement of the portal area and bridging between adjacent portal areas, resulting in pseudolobe formation. G = Glisson sheath, X = year of the patient’s death.

A liver biopsy performed in year X−6 revealed histological findings such as steatosis, inflammation, fibrosis, ballooning of hepatocytes, Mallory–Denk bodies, and pericellular fibrosis (Fig. 3A–D). Based on these histopathological features, the patient was diagnosed with NASH.

The liver was histopathologically classified as type 4 according to the Matteoni classification,^[3]^ and the activity level was 5 according to the NASH activity score.^[4]^ Figure 3 shows the interface hepatitis which is different from the metabolic NASH (metabo-NASH).

After her death, we noticed that the FIB-4 index (as described above) (age × AST/ PLT × √ALT)^[1,2]^ could have helped to predict the chronicity of the liver injury. Thereafter, we calculated the FIB-4 index to reevaluate the liver function.

Neither antinuclear nor antimitochondrial antibodies tested positive. Based on the patient’s clinical course and histological features (details are mentioned in the Discussion section), MTX was considered the most likely cause of NASH-like liver injury. Additionally, we presumed that obesity and T2DM could have worsened liver damage. We did not realize that the immediate discontinuation of MTX was necessary for treating MTX-induced liver injury. Therefore, MTX was carefully tapered from 8 to 4 mg to prevent the relapse of RA, and her body weight was strictly controlled. However, obesity and T2DM could not be controlled; and the glycohemoglobin A1c (HbA1c) level fluctuated between 12.0% and 5.2% (data not shown). As the PLT and FIB-4 index did not decrease, we discontinued MTX. By this time, she had already received 4050 mg of MTX over 13 years. Meanwhile, etanercept was replaced with intravenous tocilizumab 8 mg/kg daily for 4 weeks. After that, her polyarthritis worsened, and a psoriatic rash appeared transiently. Subsequently, tocilizumab was replaced with 100 mg subcutaneous golimumab without MTX. The RA was moderately controlled for some time. However, the FIB-4 index did not improve (Fig. 2).

Gastroduodenal endoscopic examination performed in year X−4 showed esophageal varices. Abdominal ultrasonography performed 3 years later (X−1) revealed chronic liver disease, most likely liver cirrhosis, with fatty changes and splenomegaly. No ascites or pleural effusion was observed.

**Figure 4.**
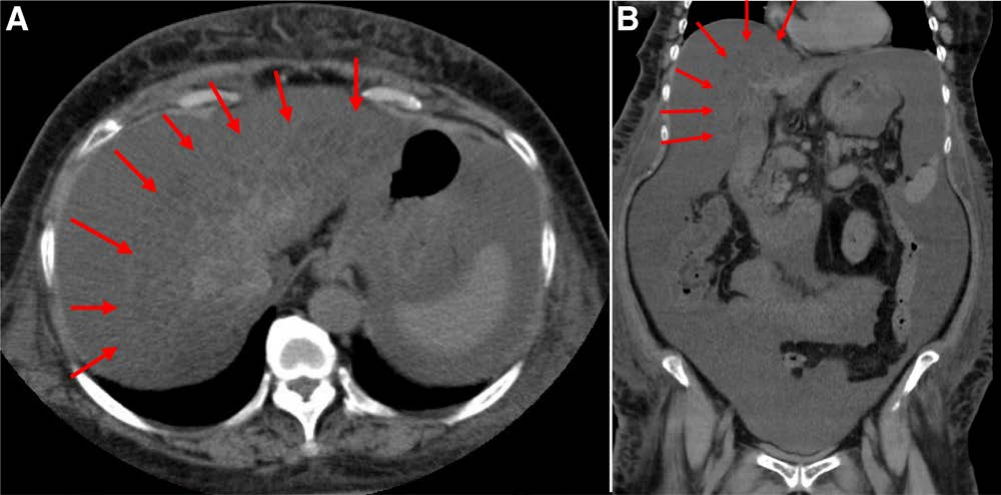
Computed tomography (CT) scan was taken during the emergency hospital visit on February 22, year X. (A) Horizontal and (B) vertical sections are shown. Arrows show the liver margins. The liver had severe atrophy and fatty changes with a mottled pattern. In addition, a large amount of ascites was visible in the abdominal cavity. X = year of the patient’s death.

Thereafter, she became bedridden, experienced steatorrhea, and developed oliguria on February 15, year X. She was brought by ambulance as an emergency patient to our hospital, and the laboratory data showed anemia (hemoglobin, 9.1 g/dL) and thrombocytopenia (PLT, 6 × 10^4^/μL), as shown in Table 1. Blood tests showed hyperbilirubinemia (total bilirubin, 6.7 mg/dL; direct bilirubin, 4.0 mg/dL). High and low-density lipoprotein cholesterol levels were 15 mg/dL and 21 mg/dL, respectively, and the HbA1c level was 5.0%, which were all abnormally low. Furthermore, the serum ammonia level was elevated to 151 μg/dL. This finding suggested that the patient was in the final stage of hepatic failure. An immediate computed tomography scan demonstrated an atrophic liver with necrosis, severe steatosis, and a large amount of ascites. The outline of the liver was unclear, as shown by the arrows in Figure 4. Eventually, she died of hepatic failure 2 days after hospitalization. Unfortunately, an autopsy could not be performed.

**Table 1:** Laboratory findings at emergency admission on February 22, year X.

Peripheral blood test		Normal range				Normal range
WBC	11 800/μL	(4.0–9.0 × 10^4^)	CRP	1.12 mg/dL		(0–0.2)
RBC	341 × 10^4^/μL	(7.36–5.00 × 10^6^)	ESR	11 mm/h		(≤20)
Hb	9.1 g/dL	(12.0–16.0)				
Hct	29.00%	(33.5–45.0)	NH3	151 μg/dL		(30–80)
PLT	6 × 10^4^/μL	(15.0–35.0 × 10^4^)				
			Fasting BS	122 mg/dL		(≤110)
Biochemical test			HbA1c	5.00%		(≤6.2)
T.bil	6.7 mg/dL	(0.2–1.0)				
D.bil	4.0 mg/dL	(0–0.2)	HBs Ag			Negative
TP	6.7 d/dL	(6.7–8.3)	HBs Ab			Negative
Alb	2.47 g/dL	(≥4.0)	HBc Ab			Negative
AST	61 U/L	(13–33)	HCV Ab			Negative
ALT	34 U/L	(6–27)	Antinuclear antibody			Negative
LDH	378 U/L	(119–229)	Antimitochondrial antibody			Negative
ALP	452 U/L	(115–359)				
γGPT	26 U/L	(10–47)	Coagulation test			
Ch-E	63 U/L	(30–70)	PT%		26.0	(70–100)
			APTT		58.8 s	(27.6–34.3)
BUN	15.3 mg/dL	(8–23)	ATIII		24.90%	(83–118)
Cre	1.19 mg/dL	(0.45–1.24)	FDP		16.8 μg/mL	(0–4.9)
UA	7.8 mg/dL	(2.8–7.0)	D-dimer		13.9 μg/mL	(0–0.9)
Ca	8.0 mg/dL	(8.2–10.2)	Urinalysis			
			Protein		1+	
TC	58 mg/dL	(130–220)	Blood		±	
TG	40 mg/dL	(0–150)	WBC		>100/HPF	
HDL-C	15 mg/dL	(≥40)	Cast		10–30/LPF	
LDL-C	21 mg/dL	(70–139)				
CK	86 U/L	(45–163)				

γGPT = gamma-glutamyl transpeptidase, Alb = albumin, ALP = alkaline phosphatase, ALT = alanine aminotransferase, APTT = activated partial thromboplastin time, AST = aspartate aminotransferase, ATIII = antithrombin III, BUN = blood urea nitrogen, Ca = calcium, Ch-E = choline esterase, CK = creatinine kinase, Cre = creatinine, CRP = C-reactive protein, D.bil = direct bilirubin, ESR = erythrocyte sedimentation rate, Fasting BS = fasting blood sugar, FDP = fibrin/fibrinogen degradation product, Hb = hemoglobin, HbA1c = glycohemoglobin A1c, HBc Ab = hepatitis B virus core antibody, HBs Ab = hepatitis B virus surface antibody, HBs Ag = hepatitis B virus surface antigen, Hct = hematocrit, HCV Ab = hepatitis C virus antibody, HDL-C = high-density lipoprotein cholesterol, HPF = high power field, LDH = lactate dehydrogenase, LDL-C = low-density lipoprotein cholesterol, LPF = low power field, NH3 = ammonia, PLT = blood platelet, PT = prothrombin time, RBC = red blood cell, T.bil, = total bilirubin, TC = total cholesterol, TP = total protein, UA = uric acid, WBC = white blood cell, X = year of the patient’s death.

## 3. Discussion

The patient was diagnosed with psoriatic arthritis according to the classification criteria for psoriatic arthritis,^[5]^ based on medical history and transient presentation with psoriatic rash during the clinical course. Moreover, she met the criteria for RA.^[4–6]^ The symmetrical distribution of arthritis, radiography, and hand echography was characteristic of RA. In addition, she had no dactylitis, juxta-articular new bone formation, or nail dystrophy. The patient later presented with psoriatic rash and was seronegative; however, she was diagnosed with RA because of the typical clinical features of RA.

She was the first patient to die of hepatic failure due to NASH-like liver injury in our hospital. Before that time, we had never lost a patient with hepatic failure due to a NASH-like liver injury. Three possible reasons might have caused the patient’s death.

First, MTX was discontinued 4 years after the diagnosis of NASH-like liver injury, which might have progressed irreversibly during this period. We treated the NASH-like liver injury by reducing the MTX dose and controlling the underlying conditions, including obesity and T2DM. The FIB-4 index, calculated after the patient’s death, did not decrease after reducing the MTX dose or discontinuing MTX. Therefore, we concluded that the decision to discontinue MTX should have been made in the initial stages. Based on our experience with this patient, we suggest that MTX should be discontinued immediately for patients with MTX-induced liver injury. In addition, we recently reported that MTX-induced NASH-like liver injury (MTX-NASH) could be improved by immediate MTX discontinuation.^[2]^

Second, the obesity and T2DM were poorly controlled. Obesity and T2DM are associated with the development and exacerbation of metabo-NASH through insulin resistance. Rapid fluctuations in HbA1c levels between 12% and 5.2%, possibly due to variations in diet and exercise, might have further contributed to the progression of MTX-NASH.^[7]^

Third, she was diagnosed with severe liver injury only after the disease progressed. Severe liver damage was discovered later. We could not diagnose chronic liver injury due to MTX using routine liver function tests, such as AST/ALT, because the values were almost normal. We recently reported that the FIB-4 index, proposed as a predictor of liver fibrosis in human immunodeficiency virus/hepatitis C coinfection,^[1]^ could be used to detect MTX-NASH.^[2]^ In this previous study,^[2]^ the mean FIB-4 index + 1 standard deviation was 3.5 for patients with RA treated with MTX in outpatient clinics. The FIB-4 index of our patient was greater than the cutoff value of 3.5 to a value of 6.15, at 4 years after MTX administration (X−11).

The FIB-4 index was calculated retrospectively because its predictive properties were not known initially. The FIB-4 index suggested fibrous changes in the liver 4 years after MTX administration (X−11). The FIB-4 index gradually increased before the liver biopsy was conducted.

Japanese patients tolerate MTX less than Caucasians because of differences in metabolism.^[8]^ Furthermore, the risk of liver injury increases with the total cumulative MTX dose and alcohol consumption.^[9]^ Therefore, Japanese patients should be carefully monitored for adverse effects of MTX, such as hepatotoxicity. Despite its good efficacy, the administration of MTX has been restricted owing to concerns regarding the risk of liver fibrosis based on numerous studies since the 1960s.^[10]^ Accordingly, MTX is used for psoriasis and psoriatic arthritis. The Group for Research and Assessment of Psoriasis and Psoriatic Arthritis guidelines in 2021 recommends that MTX should not be used for fatty liver, active hepatitis B, and active hepatitis C.^[11]^

Chronic MTX hepatotoxicity in this patient was asymptomatic and could not be detected by routine liver function tests, such as AST/ALT. Hence, the FIB-4 index should be applied to data obtained from routine blood tests.

Osuga et al^[12]^ evaluated whether there were differences in histology between MTX-NASH and metabo-NASH. The study revealed mild macrovesicular steatosis, unlike the prominent ballooning degeneration. Furthermore, inflammation and fibrosis were accentuated in the portal areas with faint lobular inflammation in MTX-NASH. The findings of prominent inflammation in the portal areas (Glisson sheath [G]) and fibrosis between G and G (bridging formation) in this patient (Fig. 3) suggest that MTX is fully involved in liver damage and histological changes similar to those of NASH in this patient were mainly induced by MTX rather than obesity and T2DM. In this patient, the combination of obesity and T2DM may have exacerbated MTX-NASH and caused death. When we encounter a patient with MTX-NASH and metabo-NASH, we usually discontinue MTX immediately and try to improve metabolic syndrome. After our experience with this patient, we have not reported any other case that deteriorated because of MTX-NASH. Following the new guidelines revised by the Japanese Society of Gastroenterology and the Japan Society of Hepatology in 2020,^[13]^ this patient’s diagnosis was drug-induced liver disease similar to NASH, which might have been aggravated by metabolic syndrome.

Careful attention should be paid when treating patients with RA using MTX, especially those with an underlying metabolic syndrome that may aggravate MTX-NASH. In such cases, MTX should be discontinued as soon as possible. Simultaneously, it is mandatory to control metabolic syndrome.

## 4. Postscript

Before 2020, drug-induced NASH-like liver injury was included in the etiology of NASH in evidence-based practice guidelines for nonalcoholic fatty liver disease/NASH in Japan. In 2020, the Japanese Society of Gastroenterology and Japan Society of Hepatology revised the guidelines and removed drug-induced liver injury from the etiology of NASH.^[13]^

## Acknowledgments

The authors appreciate Ms. Y Sato, A. Ando, H Watanabe, and A. Takei for nursing this patient and Editage (www.editage.com) for English language editing.

## Author contributions

**Investigation:** Masayuki Miyata, Sho Ishiwata, Masahito Kuroda, Kazuhiro Tasaki.

**Resources:** Masahito Kuroda, Kazuhiro Tasaki.

**Supervision:** Kiyoshi Migita, Hiromasa Ohira.

**Writing – review & editing:** Masayuki Miyata.
